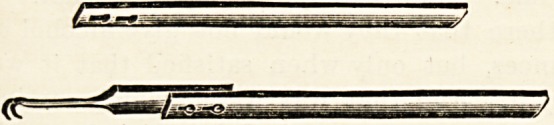# New Appliances and Things Medical

**Published:** 1901-04-06

**Authors:** 


### NEW APPLIANCES AND THINGS MEDICAL.

[We shall be glad to receive, at our Office, 28 & 29 Southampton Street, Strand, London, W.C., from the manufacturers, specimens of all new preparations andappliances which may be brought out from time to time.]

### NEW UTERINE TENACULUM.

(Reynolds & Branson, Limited, 13 Briggate, Leeds.)

This new uterine tenaculum is the invention of Mr. Alexander Duke, and has for its object the freeing of the hand, which is usually employed holding the handle of the instrument, and further than this it also holds the speculum in position. The illustration shows that the instrument is composed of two separate parts; the upper part is rigid, the lower part, the handle, is made of flexible metal, which can be bent and hooked round the lip of the speculum. The value of the invention will be chiefly appreciated in private practice where an assistant is frequently unavailable at small operations on the cervix and uterus.

### ABBEY'S EFFERVESCENT SALT.

(Abbey Effervescent Salt Co., London.)

This excellent saline preparation deserves the attention of the medical profession. From our examination we are satisfied that it is a simple mixture of mineral salts without the addition of any deleterious or harmful substance. It effervesces freely when dissolved in water, and constitutes a most agreeable and refreshing beverage. For neutralising constitutional acidity, or the acidity which arises from over indulgence in food or alcohol it is a very valuable corrective. There can be no question that the majority of chronic complaints, such as rheumatism, gout, and indigestion, can be more satisfactorily treated by moderation in diet and a daily dose of a mild saline aperient, than by any other more vigorous method. The so-called acid intoxications, whether responsible or not for the majority of the symptoms accompanying these conditions, certainly intensify the attendant discomforts, and can readily be ameliorated and neutralised by a judicious combination of carbonates and other organic salts. There is an old superstition that the constant use of alkalies is lowering to the system. There is no scientific foundation for this belief whatsoever, when the salts are properly combined so as to contain no great excess of sodium, potassium, or calcium. This object has evidently been kept in view in the preparation of Abbey's Salt. These may be taken two or three times a week, or even oftener if there is a tendency to sluggishness of the bowels.

### CACODYLATES OF IRON AND SODIUM.

(E. Merck, Darmstadt, and 16 Jewry Street, London, E.C.)

These salts are combinations of cacodylic acid with iron and sodium respectively. Cacodylic acid is now largely used in preference to arsenious acid, since it is an organic substitution acid (dimethel arsenic acid) and better tolerated by the system. It may be used in all cases in which the administration of arsenic is indicated, and for anæmia, neurasthenia, and other atonic conditions, clinical experience has proved it to be a most valuable drug. In addition to the above salts cacodylic acid can be combined with mercury, guaiacol, quinine, lithium, calcium, and other metals, according to the indication of each particular case.

## Figures and Tables

**Figure f1:**